# Effectiveness of De-Escalation in Reducing Aggression and Coercion in Acute Psychiatric Units. A Cluster Randomized Study

**DOI:** 10.3389/fpsyt.2022.856153

**Published:** 2022-04-07

**Authors:** Andreja Celofiga, Blanka Kores Plesnicar, Jure Koprivsek, Miha Moskon, Dominik Benkovic, Hojka Gregoric Kumperscak

**Affiliations:** ^1^Department of Psychiatry, University Medical Centre Maribor, Maribor, Slovenia; ^2^Faculty of Medicine, University of Maribor, Maribor, Slovenia; ^3^University Psychiatric Clinic Ljubljana, Ljubljana, Slovenia; ^4^Faculty of Medicine, University of Ljubljana, Ljubljana, Slovenia; ^5^Faculty of Computer and Information Science, University of Ljubljana, Ljubljana, Slovenia; ^6^Faculty of Natural Sciences and Mathematics, University of Maribor, Maribor, Slovenia; ^7^Child and Adolescent Psychiatry Unit, University Medical Centre Maribor, Maribor, Slovenia

**Keywords:** aggression, restraint, de-escalation training, incidence, psychiatry, acute ward

## Abstract

**Objective:**

Most guidelines for the management of aggressive behavior in acute psychiatric patients describe the use of de-escalation as the first-choice method, but the evidence for its effectiveness is inconsistent. The aim of the study was to assess the effect of verbal and non-verbal de-escalation on the incidence and severity of aggression and the use of physical restraints in acute psychiatric wards.

**Methods:**

A multi-center cluster randomized study was conducted in the acute wards of all psychiatric hospitals in Slovenia. The research was carried out in two phases, a baseline period of five consecutive months and an intervention period of the same five consecutive months in the following year. The intervention was implemented after the baseline period and included training in verbal and non-verbal de-escalation techniques for the staff teams on experimental wards.

**Results:**

In the baseline study period, there were no significant differences in the incidence of aggressive behavior and physical restraints between the experimental and control groups. The incidence rates of aggressive events, severe aggressive events, and physical restraints per 100 treatment days decreased significantly after the intervention. Compared to the control group, the incidence rate of aggressive events was 73% lower in the experimental group (IRR = 0.268, 95% CI [0.221; 0.342]), while the rate of severe events was 86% lower (IRR = 0.142, 95% CI [0.107; 0.189]). During the intervention period, the incidence rate of physical restraints due to aggression in the experimental group decreased to 30% of the rate in the control group (IRR = 0.304, 95% CI [0.238; 0.386]). No reduction in the incidence of restraint used for reasons unrelated to aggression was observed. After the intervention, a statistically significant decrease in the severity of aggressive incidents (*p* < 0.001) was observed, while the average duration of restraint episodes did not decrease.

**Conclusion:**

De-escalation training is effective in reducing the incidence and severity of aggression and the use of physical restraints in acute psychiatric units.

**Clinical Trial Registration:**

[www.ClinicalTrials.gov], identifier [NCT05166278].

## Introduction

Aggressive behavior is a common and serious problem in acute psychiatric settings. A meta-analysis of 35 studies found a pooled prevalence of aggressive behavior in patients admitted to acute psychiatric wards ranging from 3 to 44% in high-income countries (1). In European acute psychiatric wards for adult patients with mixed diagnoses, the average aggression incidence rate is 9.3 events per patient per year (2). A significant proportion of patients with aggressive behavior engage in recurrent aggressive behavior, with less than 15% being responsible for 50% of incidents or even more (3, 4).

Various forms of aggressive behavior are the most common reason for using coercive measures (5–7), which, due to ethical dilemmas and clinical consequences, have been among the most controversial topics since the beginning of modern psychiatry (8, 9). Clinical guidelines for the management of aggressive behavior recommend the use of physical restraints only as a last resort to prevent serious injury to patients and staff (10–12). However, data from seventeen European countries revealed that the use of physical restraints is the most common intervention in the management of aggressive behavior, followed by seclusion, application of pharmacotherapy, talking with the patient, and the use of de-escalation techniques (13).

The frequency, type, and duration of coercive measures used in different countries vary significantly due to a variety of factors, including legal provisions, cultural differences, tradition, policies, and differences between health systems (14). There is a heterogeneous pattern of legislation and clinical practice in the use of coercive methods in European countries, but the link between law and practice indicates that more restrictive legislation has resulted in more restrictive practices (15). The prevalence of the use of restraints in patients within psychiatric wards in different countries is estimated at between 3.8 and 20% (16). A comparison of datasets from four similar European countries revealed that the percentage of patients subjected to restraints ranged from 4.5 to 9.4%, with a monthly rate of 17–21 events per 100 admissions, and an average of three restraints per patient (17). Some studies provide data on different types of restraint (manual, mechanical, or physical, the latter usually refers to mechanical). A review of 122 studies on the incidence of aggressive behavior among hospitalized psychiatric patients shows that manual restraint was used in 34% of aggressive incidents, seclusion in 21%, while data on the use of mechanical restraints were available in only a few studies, ranging from 13 to 46% of incidents (18). A summary of the use of coercive measures in involuntarily hospitalized patients in 10 European countries showed that coercive measures were used in 38% of patients, with forced medication being the most commonly used intervention (56%), followed by restraint (36%) and seclusion (8%) (6). Physical restraints are frequently used in elderly patients to prevent falls, but in such cases, only a partial restriction with belts, such as a restriction of only the upper extremities, is more commonly used (19, 20). The more frequent use of restraints in some countries is also related to the lack of adequate room capacity for seclusion (6).

The duration of coercive measures used varies significantly across countries, ranging from less than a half-hour to seventy days, with longer restraints primarily in psychogeriatric and forensic units (14, 19, 21). Large discrepancies in the incidence and time interval of physical restraint use may also reflect restrictive legislation on the involuntary administration of psychopharmacotherapy in some countries, resulting in higher rates of aggressive incidents and the use of restraints (14, 15).

Guidelines for the management of aggressive behavior in patients with mental disorders recommend the use of de-escalation as the method of first choice (12, 22, 23), however, uncertainty remains about the effectiveness of de-escalation due to limited and inconsistent evidence (24–26). Several observational studies have shown a reduction in the incidence of aggression or coercive measures following the implementation of organizational and structural interventions in the ward environment, staff, and patient care (27). De-escalation techniques were included in several experimental studies on the effectiveness of non-pharmacological management of aggression. Putkonen et al. found a statistically significant reduction in the incidence and duration of restraints after the use of multimodal intervention, which included de-escalation (28), while Abderhalden et al. and van de Sande et al. reported reduced aggression and coercion in acute psychiatric wards after routine structured risk assessment (29, 30). Most research on the effectiveness of de-escalation has certain limitations (24–26). Often, several interventions are simultaneously included in the research, which makes it difficult to assess the impact of an individual method. Psychiatric wards often differ in size, structure, and psychopathology, with some studies involving small samples within a specific psychiatric population, making it impossible to generalize results (31, 32).

Two Cochrane reviews have highlighted the lack of relevant research on the effectiveness of de-escalation techniques for managing aggression and agitation (26, 33). A review covering 345 studies found no randomized controlled trial evaluating the effects of de-escalation in psychosis-induced aggression (26), while the second review of 6,637 citations (33) found only one study by Deudon et al. conducted in non-psychotic patients with aggressive behavior in nursing homes (34). The authors emphasized that there is still insufficient evidence to determine the effectiveness of de-escalation for managing aggressive behavior (33). A recent study published by Ye et al. found a reduction in the frequency and duration of physical restraints after de-escalation training in wards at one large psychiatric hospital (35).

In line with the recommendations for greater emphasis on the use of de-escalation prior to the introduction of coercive measures conveyed by *the European Committee for the Prevention of Torture and Inhuman or Degrading Treatment or Punishment* during visits to several Slovenian psychiatric hospitals (36), we conducted de-escalation training for staff in all psychiatric hospitals. During the training, all hospitals were included in a cluster randomized study to evaluate the effectiveness of de-escalation in managing aggressive behavior.

## Materials and Methods

### Study Design

**FIGURE 1:**
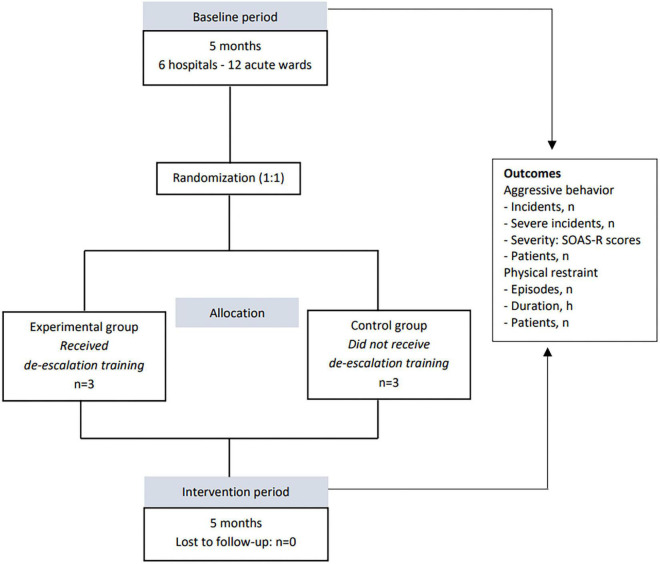
Study design.

A multi-center, cluster randomized controlled trial was conducted in all psychiatric hospitals in Slovenia. The research was conducted in two phases, a baseline period of five consecutive months in 2018 and an intervention period during the same five consecutive months in 2019. The trial was prepared in accordance with the Consort guidelines (37). The study design is shown in Figure 1.

### Settings and Participants

In Slovenia, six psychiatric hospitals, all belonging to the public sector, provide inpatient psychiatric treatment for two million inhabitants. There are two acute psychiatric wards in each hospital, one for male and one for female patients. The bed capacities of individual wards are between 14 and 25 patients, who are accommodated in rooms for one to three patients. All wards are closed and are comparable in hospital policy, patient psychopathology, and therapeutic approach. The criteria for admission and the use of coercive measures are defined and regulated by the Mental Health Act (MHA) (38). The use of coercive measures in psychiatric inpatient settings is permitted only within acute wards.

### Randomization

At the end of the baseline period, the hospitals were randomly allocated to either the experimental or the control group. Randomization was performed at the level of hospitals, not wards, to avoid possible transmission of the effect of the intervention among psychiatric hospital staff. The acute wards in an individual psychiatric hospital cannot be completely isolated, as part of the staff covers both wards in the afternoon and night shifts. Hospital staff from the experimental group were trained in verbal and non-verbal de-escalation techniques, while in the control group, the usual treatment was provided.

### Intervention

The intervention was implemented after the baseline period and included training in verbal and non-verbal de-escalation techniques for the staff teams on experimental wards. The training course was prepared according to the recommendations of the Beta Project of the American Association for Emergency Psychiatry (22), the World Health Organization Quality Rights training to act, unite and empower for mental health (39) and a handbook for the use of de-escalation skills in a hospital setting by Amdur et al. (40). (Guidelines from the Beta Project of the American Association for Emergency Psychiatry are available on: https://westjem.com/articles/verbal-de-escalation-of-the-agitated-patient-consensus-statement-of-the-american-association-for-emergency-psychiatry-project-beta-de-escalation-workgroup.html). The de-escalation training was 16 h duration in total. The first part of the training focused on a review of theoretical backgrounds concerning aggressive behavior with an emphasis on risk factors for aggression that can be modified (situational, interactive) and de-escalation techniques. The second part consisted of a workshop with a presentation of different de-escalation techniques, video material and a role play. For educational purposes, we have prepared a manual, that covers the following topics: definition of de-escalation, situations in which it is useful, the importance of safe conditions for de-escalation, communication, non-verbal and verbal de-escalation techniques, identifying one’s own non-verbal communication, coping with emotional responses and techniques that are better avoided. De-escalation training protocol is shown in Table 1. Each trainee received a short handbook and a list of verbal and non-verbal approaches. Two repetitions of the training were conducted at each hospital in the experimental group, and these were attended by all members of the staff teams. During the study, there were no other changes regarding the ward, staff, patient management, or initiatives to reduce aggression or restraints.

**TABLE 1:** De-escalation training protocol.

I. Background theory
1. Aggressive behavior in persons with mental disorders
The prevalence of aggressive behavior
Risk factors for aggressive behavior
Characteristics of aggressive behavior
Consequences of aggressive behavior
2. Communication
Basic principles
Verbal communication
Non-verbal communication
3. De-escalation
What is de-escalation
When should de-escalation be used
When to avoid using de-escalation
4. Establishing a safe environment for de-escalation
5. Non-verbal de-escalation techniques
Personal space
Body posture
Eye contact
Face mimic
Movement and gestures
Touch
Speech (tone of voice, volume, and speed of speech)
6. Verbal de-escalation techniques
Establishing verbal contact (one person, respectful communication, honesty)
Concise and clear communication (short sentences, repeating, avoiding complex questions)
Active listening (short non-verbal responses, reflection and paraphrasing)
Identifying patients’ wants and feelings
Limits and rules-setting
Offering choices and alternatives
Time out
Creating an alliance
Agree or disagree (finding something on which to agree)
Distracting, changing subjects
Taking responsibility
Withdrawal strategy
Humor
Praise, apologies, use of words please and thank you
Debriefing
7. Techniques that are better avoided: insincerity, false promises, provocative communication, interruption during speech, use of excessively professional terms, minimizing patient problems, “mind reading,” “why” questions, authoritative approach, global phrases (calm down…) etc.
**II. A workshop: presentation of clinical cases and video material, role-playing (based on real scenarios from clinical practice)**
1. Demonstration of various de-escalation techniques
2. Appropriate and inappropriate approaches to dealing with an agitated patient
3. Using non-verbal and verbal de-escalation techniques
4. Recognizing and managing one’s own emotional responses

In the wards of the control group, treatment as usual was provided. There was no de-escalation training for the staff from control group during the study, however, the training was scheduled for the period after the completion of the study. Regular annual training on the use of coercive measures was conducted as usual in all hospitals.

### Outcome Measures

The primary outcome was the number of aggressive incidents and the restraint episodes for the baseline and intervention periods, while the severity of aggressive incidents and the duration of restraints were a secondary outcome. The number of patients with aggressive behavior and restrained patients was also obtained. It should be noted that the term “patients” refers to treatment episodes or cases. Because of readmissions, the number of patients is usually lower than the number of cases. Data on aggressive incidents and severity were recorded by the revised Staff Observation Aggression Scale (SOAS-R) (41). The original version of the SOAS-R was translated into Slovenian and re-translated into English, which was evaluated by independent experts and approved by the authors of the original SOAS-R (Palmstierna T, Nijman H). The SOAS-R defines aggression as any form of verbal, non-verbal, or physical behavior that threatens or has harmed the patient himself, others, or property (41, 42). The severity of incidents was measured using the SOAS-R scoring system, ranging from 0 to 22 points (41). Incidents with a severity of nine or more points on the SOAS-R score were regarded as severe incidents, as recommended by the authors of the SOAS-R (personal communication) and used in most research in recent years (30, 43, 44). The severity of aggressive incidents was defined categorically as the presence (SOAS-R ≥ 9 points) or the absence of a severe aggressive incident (SOAS-R < 9 points) and numerically with the number of SOAS-R points. The scale was introduced into regular practice in all wards 2 months before the study, as recommended by the authors (2). Short training on how to complete the scale was provided for the staff. Data on the use and duration of restraints were obtained from the documentation that is standardized in all hospitals in accordance with the MHA (38). Slovenian MHA defines two types of coercive measures: seclusion and physical restraints with belts (PR). PR are traditionally the primary coercive measure in Slovenia, while seclusion is rarely used. The episode of PR in our study represents each individual restraint and covers the period from introduction to discontinuation. Data were obtained for all PR and for PR, where different types of aggressive behavior were defined as the reason for their introduction. Data on the number of patients and characteristics of patients and wards were obtained from hospital registries and medical records, and a standardized data acquisition form was used in all hospitals. One of the researchers collected data from the SOAS-R forms every month in all psychiatric hospitals. Underreporting of incidents was checked by regular hospital visits and comparison with the incident report records, which were a well-established and unified method of incident monitoring in all psychiatric hospitals. The incident report is a form used to monitor adverse events, including aggressive behavior in patients. Reports are regularly recorded by nurses in the case of adverse events. Sociodemographic and clinical data of patients were summarized at the end of each study period from the hospital documentation. During the intervention period of the study, data were obtained separately for the experimental and control groups, and in the baseline period, data were arranged according to which group the individual hospitals were allocated to after randomization. Data on bed capacities and the number of nursing staff were also obtained.

### Statistical Analysis

The incidence rates were expressed as the proportion of patients with at least one incident and as incident rates per 100 treatment days, with a 95% confidence interval (95% CI). The incidence rate ratio (IRR) for an aggressive incident, severe aggressive incident, and PR between the experimental and control groups was calculated for the baseline and intervention periods as recommended by Rothman and Kirkwood (45, 46), then the change in IRR between the baseline and intervention period was calculated. The risk ratio (RR) of aggressive behavior and the use of restraints with a 95% CI in patients between the experimental and control groups was calculated for the baseline and intervention periods using a generalized linear model. The difference in the incidence of aggression and restraints between the experimental and control groups was tested using the chi-square test and the Wald *z*-test. The severity of aggressive incidents was presented as the mean value of SOAS-R points, median and interquartile range. The difference in the severity of incidents between groups in each phase of the study was calculated using the Mann-Whitney U test. Differences in the duration of restraint episodes between the experimental and control groups were presented by comparing the sum of restraint hours with the sum of hospital hours for all patients in each group and the study period. Then the risk ratios for the restraint hour between the experimental and control groups were calculated for each study period.

The effect of the intervention on the number of incidents, controlling for patient characteristics, was examined using regression analysis. Based on the values of Akaike (AIC) and Bayesian information criteria (BIC), the negative binominal regression model was selected (47). Several regression analyses were performed for the baseline and intervention periods, as well as for the control and experimental groups separately. Predictors of the number of incidents included intervention and patient characteristics or study phase and patient characteristics, with the logarithm of the number of patient days included as an offset. Patients’ characteristics were selected based on regression analyses to identify predictors, and characteristics that differed between groups were included.

The characteristics of aggressive patients and aggressive incidents were analyzed using descriptive statistical methods. Categorical variables were presented as frequencies and percentages. The distribution of the quantitative variables was checked using the Kolmogorov-Smirnov or Shapiro-Wilk test. Quantitative variables were presented using the mean and standard deviation or median and interquartile range. Differences in patient characteristics between groups in the baseline and intervention periods were examined using the Pearson chi-square test, and the Mann-Whitney U test due to non-normal distribution of quantitative variables. Statistical analysis was performed using the IBM Statistical Package for the Social Sciences, version 25 (48) and Python together with the statsmodels module (49). The significance level of all statistical tests was determined at *p* < 0.05, two-sided.

## Results

A total of 6,401 patients were included in the study, 3,190 in the baseline period of the study, which represents a total of 30,895 treatment days, and 3,211 in the intervention period, accounting for 29,131 treatment days. It must be noted that the term “patients” refers to treatment episodes or cases. Because of readmissions during the study, the actual number of patients was lower (5,615) than the number of treatment episodes (6,401). During the study periods, there were no differences in patients between the experimental and control groups in most sociodemographic and clinical characteristics (Table 2). However, in the control group, compared to the experimental, the proportion of involuntary admitted patients was higher in both study periods, and the differences were statistically significant (baseline: 22.2 and 9.3%, *p* < 0.001, intervention: 21.9 and 9.2%, *p* < 0.001). There was also a significant difference between the groups in the duration of hospitalization, which was longer in the experimental group in both study periods (baseline: experimental—mean 11.83, *SD* 14.190, control—mean 8.30, *SD* 12.709, *p* < 0.001; intervention: experimental—mean 11.06, *SD* 12.355, control—mean 7.64, *SD* 12.205; *p* < 0.001). In the baseline study period, a significantly higher proportion of patients with a comorbid diagnosis of F1 was observed in the control group than in the experimental group (22.7 and 17.4%, *p* < 0.001). During the study, 1,544 aggressive incidents involving 780 patients (12.2%) and 1,305 restraint episodes involving 699 patients (10.9%) were recorded.

**TABLE 2:** Patient and ward characteristics.

	Experimental group	Control group	*p*-value
**Baseline period**			
Patients, *n*	1,251	1,939	
Treatment days, *n*	14,796	16,099	
**Patient characteristics**			
Gender, male: *n* (%)	689 (55.1)	1,013 (52.2)	0.117
Age, years: mean (*SD*), median, range	48.36 (17.026), 46.00, 16–97	49.18 (19.100), 49.00, 14–96	0.330*
Hospitalization lenght, days: mean (*SD*), median, range	11.83 (14.190), 7.00, 1–146	8.30 (12.709), 4.00, 1–153	< 0.001*
Involuntary hospitalization, *n* (%)	116 (9.3)	430 (22.2)	< 0.001
Main diagnosis, *n* (%)			
F0 F1 F2 F3 F6 Other	157 (12.5) 267 (21.3) 417 (33.3) 200 (16.0) 34 (2.7) 176 (14.1)	229 (11.8) 377 (19.4) 701 (36.2) 357 (18.4) 48 (2.5) 227 (11.7)	0.532 0.192 0.103 0.078 0.673 0.050
Comorbid F1, *n* (%)	218 (17.4)	441 (22.7)	< 0.001
Comorbid F6, *n* (%)	127 (10.2)	177 (9.1)	0.336
**Ward characteristics**			
Number of beds: mean (*SD*), median, range	20.33 (3.615), 18.00, 18–25	18.33 (3.011), 18.50, 14–22	0.506*
Nursing staff: mean (*SD*), median, range	14.17 (0.983), 14.50, 13–15	15.17 (2.483), 14.50, 13–18	0.615*
**Intervention period**			
Patients, *n*	1,347	1,864	
Treatment days, *n*	14,893	14,238	
**Patient characteristics**			
Gender, male: *n* (%)	726 (53.9)	984 (52.8)	0.535
Age, years: mean (*SD*), median, range	48.27 (17.462), 48.00, 15–99	49.43 (17.905), 48.00, 14–99	0.067*
Hospitalization lenght, days: mean (*SD*), median, range	11.06 (12.355), 7.00, 1–100	7.64 (12.205), 3.00, 1–153	< 0.001*
Involuntary hospitalization, *n* (%)	124 (9.2)	409 (21.9)	< 0.001
Main diagnosis, *n* (%)			
F0 F1 F2 F3 F6 Other	173 (12.8) 289 (21.5) 463 (34.4) 241 (17.9) 33 (2.4) 148 (11.0)	221 (11.9) 395 (21.2) 696 (37.3) 326 (17.5) 34 (1.8) 192 (10.3)	0.400 0.857 0.084 0.768 0.221 0.532
Comorbid F1, *n* (%)	264 (19.6)	402 (21.6)	0.175
Comorbid F6, *n* (%)	156 (11.6)	191 (10.2)	0.229
**Ward characteristics**			
Number of beds: mean (*SD*), median, range	20.33 (3.615), 18.00, 18–25	18.33 (3.011), 18.50, 14–22	0.506*
Nursing staff: mean (*SD*), median, range	14.17 (0.983), 14.50, 13–15	15.17 (2.483), 14.50, 13–18	0.615*

SD, standard deviation; F0, Organic, including symptomatic, mental disorders, F1, Mental and behavioral disorders due to psychoactive substance use, F2, Schizophrenia, schizotypal and delusional disorders, F3, Mood disorders, F6, Disorders of adult personality and behavior, p-values*, Mann Whitney U-test; other p-values, Chi square test.

### Incidence of Aggressive Behavior

The number of patients with aggressive behavior and the number of incidents in the experimental group decreased from baseline to the intervention period of the study compared to the control group. During the study, we recorded 922 aggressive incidents in the baseline period and 622 in the intervention period. The incidence rate ratio (IRR) per 100 treatment days showed a comparable incidence of all aggressive and severe aggressive events between the two groups in the baseline period, while in the intervention period, the incidence rate of aggressive events in the experimental group was 27% of the incidence in the control group, and the incidence rate of severe events was only 14% of the incidence rate in the control group. Absolute numbers of incidents and further data on the incidence rate and the incidence rate ratio in the baseline and intervention periods are provided in Table 3.

**TABLE 3:** Aggression and restraint rates during baseline and intervention periods.

	Experimental group		Control group		IRR [95% CI] Experimental/Control	*p*-value
	N	Rate [95% CI]	N	Rate [95% CI]		
**Treatment days**						
Baseline	14,796		16,099			
Intervention	14,893		14,238			
**Aggressive incidents**						
Baseline	441	2.981 [2.715; 3.272]	481	2.988 [2.732; 3.267]	0.998 [0.877;1.135]	*
Intervention	136	0.913 [0.772; 1.080]	486	3.413 [3,123; 3.731]	0.268 [0.221; 0.324]	**
Change		-69.4%		+ 14.2%	-73.2%	
**SOAS-R ≥ 9**						
Baseline	334	2.257 [2.028; 2.513]	349	2.168 [1.952; 2.408]	1.041 [0.896; 1.210]	*
Intervention	55	0.369[0.284; 0.481]	370	2.599 [2.347; 2.877]	0.142 [0.107;0.189]	**
Change		-83.7%		+19.9%	-86.4%	
**Restraints**						
Baseline	377	2.548 [2.303; 2.819]	394	2.447 [2.217; 2.701]	1.041 [0.904: 1.199]	*
Intervention	192	1.289 [1.119; 1.485]	342	2.402 [2.160; 2.671]	0.537 [0.450; 0.640]	**
Change		- 49.4%		- 1.8%	- 48.4%	
**Restraints duration, h**						
Baseline	3,290	0.926 [0.895; 0.959]	5,798	1.501 [1.462; 1.540]	0.617 [0.592; 0.644]	**
Intervention	2,130	0.596 [0.571; 0.622]	6,586	1.927 [1.881; 1.974]	0.309 [0.294; 0.325]	**
Change		-35.6%		+ 28.4%	- 49.9%	

IRR, Incidence rate ratio; p, statistical difference for the incidence rate between the experimental and control groups in each study period; * no statistically significant difference (p-value of > 0.05); ** p-value of < 0.001.

The risk ratio for aggression in a patient did not show a statistically significant difference between the experimental and control groups in the baseline period (RR = 1.087, 95% CI [0.909;1.300], *p* = 0.363), but in the intervention period, the risk of aggression in a patient was 55% lower in the experimental group compared to the control (RR = 0.446, 95% CI [0.353; 0.562], *p* = 0.000). In the baseline period, we did not confirm a statistically significant difference in the risk of severe aggression in a patient between the experimental and control groups (RR = 1.086, 95% CI [0.889; 1.326], *p* = 0.421), while the risk in the experimental group in the intervention period was only 29% of the risk in the control group (RR = 0.294, 95% CI [0.215; 0.402], *p* = 0.000). Data on aggression in patients during baseline and intervention study periods are provided in the supplementary material (Supplementary Table 1). The proportion of patients with recurrent aggressive incidents in the experimental group decreased from 7.6 to 1.9% (*p* = 0.000) from the baseline to the intervention period, while the proportion of these patients did not change significantly in the control group (baseline 4.7%, intervention 5.0%; *p* = 0.670).

Regression analysis did not show a significant effect of the intervention (experimental group) on the number of aggressive incidents during the baseline study period (β = –0.082, *p* = 0.403, model significance: LLR *p*-value < 0.0001, likelihood ratio test with the null model), while in the intervention period the effect was negative and statistically significant (β = –1.150, *p* < 0.0001, model significance: LLR *p*-value < 0.0001). Both phases showed a positive effect of involuntary hospitalization (baseline: β = 0.922, *p* < 0.0001, intervention: β = 0.989, *p* < 0.0001), the main diagnosis of F6 (baseline: β = 0.713, *p* = 0.007, intervention: β = 1.179, *p* < 0.0001), the comorbid F6 diagnosis (baseline: β = 0.339, *p* = 0.026, intervention: β = 0.439, *p* = 0.002) and a negative effect of patient age (baseline: ß = –0.015, *p* < 0.0001, intervention: ß = –0.008, *p* = 0.043). The main diagnoses of F0 and F1 showed a significant positive effect in the baseline period (F0: β = 0.796, *p* < 0.0001, F1: β = 0.644, *p* ≤ 0.0001), while the effect of the F2 diagnosis was negative and significant in the intervention period (β = –0,368, *p* = 0.006). The effect of comorbid diagnosis of F1 was statistically insignificant in both phases.

In the control group, a significant effect of the intervention study period on the number of incidents in the positive direction was observed (β = 0.182, *p* = 0.033, model significance: LLR *p*-value < 0.0001), while in the experimental group, regression analysis showed a statistically significant negative effect of the intervention study period (β = –0.904, *p* < 0.0001, model significance: LLR *p*-value < 0.0001). In both groups, there was a significant positive effect of involuntary hospitalization (control: β = 0.933, *p* < 0.0001, experimental: β = 0.925, *p* < 0.0001), diagnosis of F6 (control: β = 0.612, *p* = 0.012, experimental: β = 1.555, *p* < 0.0001), diagnosis of F1 (control: β = 0.334, *p* = 0.020, experimental: β = 0.690, *p* = 0.001), and comorbid diagnosis of F6 (control: β = 0.353, *p* = 0.008, experimental: β = 0.469, *p* = 0.006). The experimental group showed a negative effect of female gender (β = –0.329, *p* = 0.012) and a positive effect of comorbid F1 diagnosis (β = 0.403, *p* = 0.007), whereas the control group showed a negative effect of patient age (β = –0.015, *p* < 0.0001), F2 diagnosis (β = –0.303, *p* = 0.008) and a positive effect of F0 diagnosis (β = 0.821, *p* < 0.0001). The results of the regression analyses are shown in the supplementary material (Supplementary Tables 2, 3).

### Incidence of Physical Restraint

**FIGURE 2:**
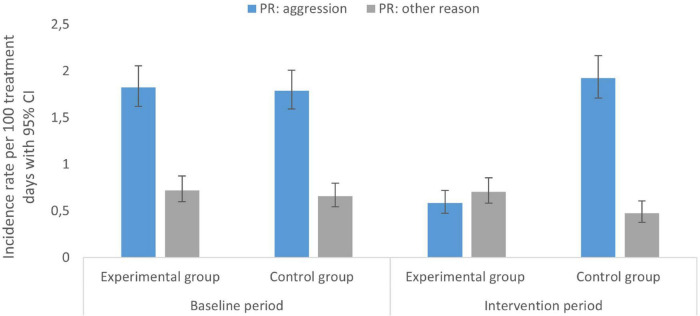
Physical restraint (PR) rates during baseline and intervention periods.

A significant reduction in the risk ratio of patients with PR between the experimental and control groups was observed from the baseline (RR = 1.040, 95% CI [0.859; 1.259], *p* = 0.687) to the intervention period (RR = 0.650, 95% CI [0.518; 0.815], *p* = 0.000), revealing the risk in the experimental group in the intervention period was 65% of the risk in the control group. Data on the use of PR in patients during baseline and intervention study periods are provided in the supplementary material (Supplementary Table 1). The proportion of patients in whom PR was used due to aggression was comparable between the two groups in the baseline period (9.9 and 8.7%, *p* = 0.253), while in the intervention period it was statistically significantly lower in the experimental group (4.9 and 9.7%, *p* = 0.000). There was no statistically significant difference between the groups in the proportion of patients with PR for other reasons, either in the first (2.5 and 3.2%, *p* = 0.238) or in the second phase of the study (2.5 and 1.8%, *p* = 0.140). Data on the incidence rate and the incidence rate ratio of PR in the baseline and intervention periods are provided in Table 3. Analysis of PR episodes according to the reason for the introduction (aggression or other causes), showed a comparable incidence rate of PR due to aggression between the two groups in the baseline period, while after the intervention, the incidence rate in the experimental group was only 30% of the incidence in the control group (IRR = 0.304, 95% CI [0.238; 0.386], *p* = 0.000). The incidence rate of episodes of PR due to other causes was slightly higher in the experimental group than in the control group in both phases of the study (Figure 2). The proportion of patients with recurrent PR episodes decreased in the experimental group from 4.8% at baseline to 2.2% in the intervention period (*p* = 0.000), while there was no significant change in the control group.

In the baseline phase of the study, negative binomial regression analysis showed no effect of the intervention (experimental group) on the number of PR episodes (β = –0.101, *p* = 0.333) or on the number of PR episodes due to aggression (β = –0.028, *p* = 0.817), while in the intervention phase there was a statistically significant effect in the negative direction (PR-all: β = –0.648, *p* < 0.0001, PR-aggression: β = –0.936, *p* < 0.0001). In both phases, a negative effect of patient age (baseline: β = –0.014, *p* = 0.0004, intervention: β = –0.012, *p* = 0.011) and a positive effect of involuntary hospitalization (baseline: β = 1.064, *p* < 0.0001, intervention: β = 1.235, *p* < 0.0001), F0 diagnosis (baseline: β = 0.905, *p* < 0.0001, intervention: β = 0.723, *p* = 0.003) and the main diagnosis of F1 (baseline: β = 0.710, *p* = 0.0003, intervention: β = 0.539, *p* = 0.012) on the number of PR due to aggression, were observed. Female gender had a significant effect only in the baseline period (β = –0.387, *p* = 0.002), while F6 diagnosis had a significant effect in the intervention period (β = 1.659, *p* < 0.0001). Again, the effect of the comorbid F1 diagnosis was statistically insignificant in both phases.

In the control group, no effect of the study period on the number of all PR episodes or PR episodes due to aggression was observed (PR-all: β = 0.020, *p* = 0.834, PR-aggression: β = 0.145, *p* = 0.170), while in the experimental group, there was a statistically significant negative effect of the intervention study period (PR-all: β = –0.559, *p* < 0.0001, PR-aggression: β = –0.767, *p* < 0.0001). In both groups, a significant positive effect of involuntary hospitalization (control: β = 1.038, *p* < 0.0001, experimental: β = 1.272, *p* < 0.0001) and the main diagnosis of F1 (control: β = 0.657, *p* = 0.0002, experimental: β = 0.634, *p* = 0.011), and a significant negative effect of female gender (control: β = –0.238, *p* = 0.041, experimental: β = –0.445, *p* = 0.004) on the number of PR due to aggression, were shown. In the experimental group, the main diagnosis of F6 showed a significant positive effect (β = 1.678, *p* < 0.0001), while in the control group a significant effect of patient age (β = –0.016, *p* < 0.0001) and F0 diagnosis was observed (β = 1.302, *p* < 0.0001). All regression models were statistically significant (*p* < 0.0001, likelihood ratio test with the null model). The results of the regression analyses are shown in the supplementary material (Supplementary Tables 4 – 8).

### Severity of Aggressive Behavior

**FIGURE 3:**
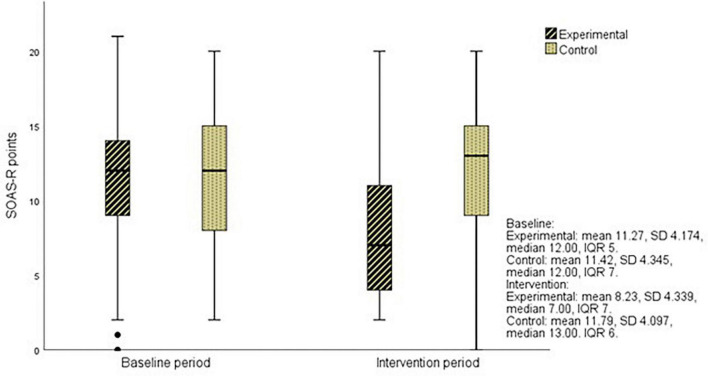
The severity of aggressive incidents in the experimental and control groups during study periods.

**FIGURE 4:**
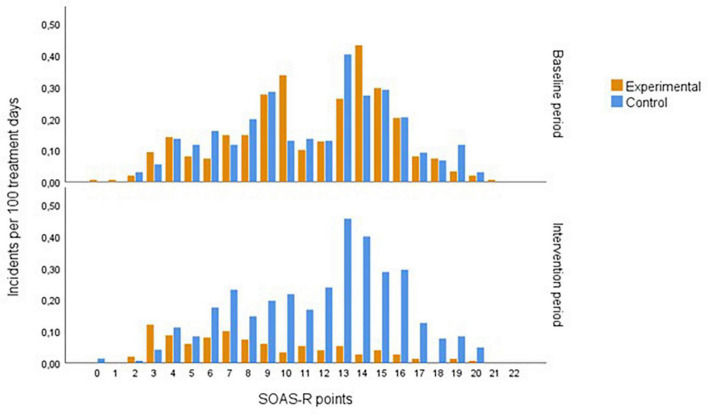
The frequency distribution of the severity of aggressive incidents during study periods.

In the baseline period, a high proportion of severe incidents (SOAS-R ≥ 9 points = 74%) was recorded, and a similar proportion (76%) remained in the control group in the intervention period, while in the experimental group, it decreased to 40%. After de-escalation training, the severity of aggressive incidents decreased significantly in the experimental group compared to the control group (*p* = 0.000) (Figure 3). The greatest decrease was observed in incidents with a severity between 8 and 18 points on SOAS-R (Figure 4). The proportion of incidents with the most serious consequences (pain, injury, need for treatment by a physician) was higher in the control group in both phases, and no significant change was recorded after the intervention within each group (experimental 5.7 and 5.9%, control 12.1 and 9.1%).

### Duration of Physical Restraint

A comparison of the number of PR hours introduced due to aggression per 100 treatment hours showed a statistically significant difference between the groups, both in the baseline period (experimental: 0.660 h per 100 treatment h, control: 0.927 h per 100 treatment h; *p* = 0.000) and in the intervention period (experimental: 0.255 h per 100 treatment h, control: 1.447 h per 100 treatment h; *p* = 0.000). After de-escalation training, a decrease in the restraint hours was observed in the experimental group but an increase was observed in the control group. The risk for a restraint hour in the experimental group in the baseline period was 71% of the risk in the control group (RR = 0.711, 95% CI [0.675; 0.749]), while it decreased to 18% in the intervention period (RR = 0.176, 95% CI [0.164; 0.189]), with 95% CI not overlapping. The risk for a restraint hour due to non-aggression-related causes was also higher in the control group compared to the experimental one in both phases of the study, and no reduction was observed after de-escalation training. The mean duration of an individual episode of PR in the baseline period was statistically significantly longer in the control group, as well as in the subgroup of PR due to aggression (experimental: mean 8.67, *SD* 9.487, median 4.38 [4.00; 7.00], control: mean 12.44, *SD* 14.043, median 10.04 [7.92; 11.58], *p* = 0.000). There was no reduction in the mean duration of an episode of PR due to aggression after the intervention, but a statistically insignificant prolongation was observed in both groups (experimental: mean 10.45, *SD* 12.587, median 7.83 [5.00, 9.00], control: mean 18.04, *SD* 35.643, median 9.83 [8.00; 11.67], *p* = 0.013).

## Discussion

This study assessed the impact of education and the use of de-escalation on the reduction in aggressive behavior and the use of PR in acute psychiatric wards. The results of the study confirm the effectiveness of de-escalation in reducing the number of patients with aggressive behavior and patients with PR, the number and severity of aggressive incidents, and the number and total duration of episodes of PR, but not the average duration of PR episodes.

The relative risk ratio for an aggressive incident decreased by 73% between baseline and the intervention period, similar to that found by Van de Sande et al., who reported a 68% reduction after a regular daily risk assessment (29). The incidence rate of severe aggressive events decreased even more after de-escalation training, as did the severity of the incidents. Contrary to the findings of Abderhalden et al. from the study of the impact of short-term risk assessment on violence (30), our study also found a significant reduction in severity within the subgroup of incidents rated above 8 points on the SOAS-R. These results may be related to the fact that the use of de-escalation, on one hand, reduced the incidence of aggressive events and, on the other hand, it prevented the progression of already perceived aggression into more severe forms. The staff were encouraged to use de-escalation as the predominant approach in patient care and to incorporate the use of de-escalation into the ward philosophy. Specific de-escalation techniques were used at high risk of aggression or in case of perceived aggressive behavior. Increased staff attention to possible re-escalation may be associated with a greater impact of the intervention in patients with recurrent incidents. The likelihood of successful de-escalation is greater if techniques that have been effective in previous incidents were used (50), which may influence the staff to respond more quickly and effectively. In the experimental group, our results showed a significant reduction in the proportion of patients with recurrent incidents from baseline to the intervention period, while no significant change was observed in the control group. In the most severe forms of aggression, de-escalation is less effective (50). The proportion of the most serious incidents did not change significantly after the intervention within each group, which is in line with the findings of Abderhalden et al. (30).

During the intervention period, a decrease in the proportion of patients with PR and the incidence rates of PR episodes was observed in the experimental group. The reduction in the incidence of coercive measures following the non-pharmacological management of aggression has been observed in several cluster randomized trials, but the comparison is limited due to the different methodology of presentation of results. Putkonen et al. reported a reduction in the proportion of hospitalization days with the use of restraints in men with severe mental disorders and in forensic patients from 30 to 15% (28), Abderhalden et al. reported a 27% reduction in the incidence of coercive measures following a short-term risk assessment using the Broset Violence Checklist (30), while Ye et al. found a statistically significant decrease in the frequency and duration of PR after de-escalation training (35). The use of seclusion and restraints is not always associated with aggressive behavior. In a 15-year follow-up of the reasons for the introduction of coercive measures, Keski-Valkama et al. found that about two-thirds of seclusion and restraints were not a consequence of violence (51). The higher the proportion of coercive measures introduced for reasons not directly related to aggression, the less reliable is the monitoring of their incidence as an indicator of the effectiveness of de-escalation. In our study, therefore, we defined an episode of PR according to the reason for its introduction and presented the incidence rate of PR episodes due to aggressive behavior as a separate group. 30% of PR episodes were related to non-aggressive reasons such as fall protection, medical treatment facilitation, application of infusions, delirium, and use at the patient’s request. Such episodes of PR are usually longer, the use of de-escalation did not affect their incidence, and the incidence rate was almost the same in both phases of the study.

The relative risk ratio for a restraint hour between the intervention and baseline periods showed a 50% reduction in RR during the intervention period, which is similar to that found by Van de Sande et al., where they reported a 45% decrease in RR (29). Considering only PR episodes due to aggression, we found a 75% decrease in RR for the restraint hour. The risk for the restraint hour due to aggression was lower in the experimental group in both study periods compared to the control group, but in the intervention period, the experimental group showed the effect of de-escalation with a further reduction, while in the control group, the risk increased. No statistically significant change in the duration of PR episodes was observed after the intervention, but PR introduced for non-aggression-related causes were statistically significantly longer than PR introduced for aggression. A considerable proportion of PR introduced for other reasons has been used in patients with organic mental disorders with confusion or delirium who, according to the literature review and survey of international trends, are associated with more than 50% of the coercive measures used (14). MHA in Slovenia requires to check the need for continued use of coercive measures every 4 h during their implementation. In practice, PR is often prolonged during the night, even though the patient is calm, to avoid the need for reintroduction, given that there are fewer staff on the night shift. In patients with severe aggression, where the introduction of PR is very stressful for the patient or staff, we often do not decide to discontinue PR as soon as the patient calms down. Occasionally, the implementation of PR is slightly modified, and the patient is only partially restricted by bands (e.g., the patient has one or both hands free). This may partially explain the fact that the duration of PR episodes itself did not change significantly after the implementation of de-escalation, but additional measures will need to be introduced to shorten the episodes of PR, especially those not due to aggressive behavior. Long-term PR that are not directly related to the patient’s behavior, but the philosophy of the staff and established clinical practice, cannot be eliminated by learning de-escalation alone. It is essential to encourage changes in the staff attitudes toward the use of coercive measures. Some researchers emphasize the importance of increasing the staff-patient ratio, setting up crisis response teams, and changes at the level of the ward environment (52). Such changes require the intensive engagement of individuals, supported by the management of the institution, both in terms of philosophy and structural and organizational changes, as well as a certain amount of time.

During the study periods, there were some significant differences in patient characteristics between the control and experimental groups. The proportion of involuntary admitted patients in the experimental group was significantly lower than in the control group, while the duration of hospitalization was significantly longer. The differences between the groups in the baseline period were comparable to the differences during the intervention period. Various studies have shown that aggressive behavior and the use of coercive measures are more common in involuntary hospitalized patients (53–57) and in patients with longer hospitalizations (58–60). Patients who are admitted involuntarily or for a longer period of time often have severe mental disorders and serious deterioration, which are also associated with a higher risk of aggression or more frequent use of coercive measures. Patients with longer hospitalizations, on the other hand, have a longer period during which incidents can occur. In both study periods, a lower proportion of patients with a comorbid diagnosis of F1 was observed in the experimental group than in the control group, while the difference was statistically significant only in the first phase. The reasons for the lower number of patients with a comorbid F1 diagnosis are not entirely clear. It could be due to the possible selection of patients with comorbid substance use disorders in control group but may also be the result of different practices for using secondary diagnoses, namely that they are often not routinely coded, particularly in the case of a secondary diagnosis of F1, relating to the harmful use of psychoactive substances. In practice, substance abuse is often described only in the patient’s documentation and in the discharge letter, and the appropriate secondary diagnosis is not used. In all regression models, there was a significant positive effect of involuntary hospitalization on the number of incidents, while the effect of comorbid F1 diagnosis was insignificant during both study periods. Hospitalization length was used in regression analyses as an offset to correct the duration at the patient level. However, all regression models showed a significant negative effect of the intervention after de-escalation training.

### Strengths and Limitations

The main advantage was the cluster randomized trial design involving all acute psychiatric wards in the country. The experimental and control wards were comparable in most characteristics, as there were no statistically significant differences in gender, age, and main diagnosis between patients in the experimental and control groups in the baseline and intervention phases of the study. There were no significant cultural and ethnic differences in the studied patient population. The intervention was easy to implement and did not require additional costs. The emphasis was on both verbal and non-verbal de-escalation techniques, which is also recommended in the guidelines of *the British Association for Psychopharmacology and the National Association of Psychiatric Intensive Care and Low Secure Units* (24). Often the emphasis is mainly on learning verbal techniques, while non-verbal skills remain in the background, even though a patient in distress receives most of the message through non-verbal communication. The training was attended by all staff members on the experimental wards, and this fostered a sense of connection and cooperation between the staff, which was often reported after the intervention. The effect of de-escalation was assessed by monitoring both aggressive incidents and PR. The episodes of PR were defined according to the reason for their introduction, as a significant proportion of these measures are not related to aggression.

There are some limitations in our study. Due to the possibility of staff transmitting the impact of the intervention within the hospital, randomization was not performed at the level of individual wards, but at the hospital level, which reduced the number of cluster randomization units and limited the power of the study. Another limitation is the fact that most of the data were obtained from the SOAS-R scale and medical records. Nursing reporting of aggressive incidents introduces the possibility of observer bias, and the lack of interrater reliability assessment limits the strength of the study. Underreporting of incidents and incorrect or partial completion of the SOAS-R may affect the outcomes, which was minimized by introducing the scale into regular clinical practice prior to the study and by checking the compliance of reported incidents with other monitoring mechanisms used in psychiatric hospitals. The study was not double-blind. However, randomization was performed after the baseline period so that baseline data could not be biased, and hospital staff were unaware of the aim and design of the study. A potential confounder could also represent the impact of the use of psychopharmacotherapy before the onset of aggressive behavior. The SOAS-R scale provided data on the pharmacotherapy used after the incident, however, other data on pharmacotherapy prescribed to patients were not obtained. There was a statistically significant difference in the proportion of involuntarily admitted patients, the comorbid diagnosis of F1, and the duration of hospitalization between the experimental and control wards. However, the use of regression analyses showed a significant effect of the intervention on the aggressive incidents and PR, controlled by the observed differences in patient characteristics.

## Data Availability Statement

The raw data supporting the conclusions of this article will be made available by the authors, without undue reservation.

## Ethics Statement

The studies involving human participants were reviewed and approved by the Slovenian National Medical Ethics Committee (0120-74/2018/4). Written informed consent for participation was not required for this study in accordance with the national legislation and the institutional requirements.

## Author Contributions

AC, JK, and BK prepared the concept and design of the study. AC and JK participated in data collection. AC drafted the manuscript. JK, BK, and HG revisited the manuscript. MM and DB participated in statistical analysis. All authors contributed to the article and approved the submitted version.

## Conflict of Interest

The authors declare that the research was conducted in the absence of any commercial or financial relationships that could be construed as a potential conflict of interest.

## Publisher’s Note

All claims expressed in this article are solely those of the authors and do not necessarily represent those of their affiliated organizations, or those of the publisher, the editors and the reviewers. Any product that may be evaluated in this article, or claim that may be made by its manufacturer, is not guaranteed or endorsed by the publisher.
