# Supplementary material for: Effectiveness of De-Escalation in Reducing Aggression and Coercion in Acute Psychiatric Units. A Cluster Randomized Study
Source: Front Psychiatry. 2022 Apr 7;13:856153. doi: 10.3389/fpsyt.2022.856153 (PMC9021532; doi:10.3389/fpsyt.2022.856153)
Supplement: Supplementary file 1 [file Data_Sheet_1.pdf]

Supplement Table 1. Aggression and use of physical restraint in patients during baseline and intervention study periods.

Patients[1]	Experimental group		Control group		RR [95% CI] Experimental/Control	*p*-value
	N	%	N	%		
All						
Baseline	1251		1939			
Intervention	1347		1864			
Aggressive behavior						
Baseline	176	14.1	251	12.9	1.087 [0.909; 1.300]	*
Intervention	86	6.4	267	14.3	0.446 [0.353; 0.562]	**
SOAS-R ≥9						
Baseline	145	11.6	207	10.7	1.086 [0.889; 1.326]	*
Intervention	45	3.3	212	11.4	0.294 [0.215; 0.402]	**
Restraints						
Baseline	155	12.4	231	11.9	1.040 [0.859; 1.259]	*
Intervention	100	7.4	213	11.4	0.650 [0.518; 0.815]	**
Restraints due to aggression						
Baseline	124	9.9	169	8.7	1.137 [0.912; 1.418]	*
Intervention	66	4.9	180	9.7	0.507 [0.386; 0.667]	**

RR, Risk ratio; p, statistical difference in the proportion of patients with aggression or restraints between the groups in each study period;
** no statistically significant difference (p-value of > 0.05); ** p-value of < 0.001.*
[1]Patients refers to treatment episodes.

Supplement Table 2. The results of the regression analysis on the number of aggressive incidents during baseline and intervention study periods.

	Coef.	Std. Err.	p	95% CI
BASELINE PERIOD				
Constant	-3,301	0,211	0,000	[-3,715; -2,887]
Intervention	-0,082	0,099	0,403	[-0,276; 0,111]
Gender	-0,083	0,102	0,416	[-0,283; 0,117]
Involuntary	0,922	0,100	0,000	[0,726; 1,119]
Age	-0,015	0,003	0,000	[-0,021; -0,008]
Comorbid F1	-0,030	0,124	0,808	[-0,273; 0,213]
Dg F0	0,796	0,186	0,000	[0,432; 1,159]
Dg F1	0,644	0,166	0,000	[0,320; 0,969]
Dg F2	-0,014	0,133	0,915	[-0,274; 0,246]
Dg F6	0,713	0,263	0,007	[0,197; 1,228]
Comorbid F6	0,339	0,153	0,026	[0,040; 0,639]
INTERVENTION PERIOD				
Constant	-3,322	0,229	0,000	[-3,770; -2,874]
Intervention	-1,150	0,124	0,000	[-1,394; -0,906]
Gender	-0,190	0,111	0,087	[-0,408; 0,028]
Involuntary	0,989	0,111	0,000	[0,772; 1,205]
Age	-0,008	0,004	0,043	[-0,015; 0,000]
Comorbid F1	0,047	0,131	0,720	[-0,210; 0,304]
Dg F0	0,205	0,207	0,323	[-0,202; 0,611]
Dg F1	0,180	0,172	0,295	[-0,157; 0,516]
Dg F2	-0,368	0,134	0,006	[-0,631; -0,106]
Dg F6	1,179	0,290	0,000	[0,610; 1,747]
Comorbid F6	0,439	0,143	0,002	[0,159; 0,720]

Supplement Table 3. The results of the regression analysis on the number of aggressive incidents in the experimental and control groups.

	Coef.	Std. Err.	p	95% CI
EXPERIMENTAL GROUP				
Constant	-3,768	0,286	0,000	[-4,328; -3,207]
Study period	-0,904	0,130	0,000	[-1,160; -0,648]
Gender	-0,329	0,131	0,012	[-0,585; -0,072]
Involuntary	0,925	0,140	0,000	[0,651; 1,198]
Age	-0,005	0,005	0,302	[-0,014; 0,004]
Comorbid F1	0,403	0,149	0,007	[0,112; 0,695]
Dg F0	-0,096	0,276	0,728	[-0,637; 0,445]
Dg F1	0,690	0,210	0,001	[0,280; 1,101]
Dg F2	0,058	0,168	0,729	[-0,272; 0,388]
Dg F6	1,555	0,328	0,000	]0,912; 2,197]
Comorbid F6	0,469	0,169	0,006	[0,138; 0,801]
CONTROL GROUP				
Constant	-3,187	0,183	0,000	[-3,545; -2,828]
Study period	0,182	0,086	0,033	[0,015; 0,350]
Gender	-0,092	0,092	0,317	[-0,273; 0,089]
Involuntary	0,933	0,087	0,000	[0,762; 1,104]
Age	-0,015	0,003	0,000	[-0,021; -0,010]
Comorbid F1	-0,188	0,113	0,095	[-0,409; 0,033]
Dg F0	0,821	0,159	0,000	[0,510; 1,132]
Dg F1	0,334	0,144	0,020	[0,052; 0,616]
Dg F2	-0,303	0,114	0,008	[-0,527; -0,078]
Dg F6	0,612	0,242	0,012	[0,137; 1,087]
Comorbid F6	0,353	0,133	0,008	[0,091; 0,614]

Supplement Table 4. The results of the regression analysis on the number of physical restraints during baseline and intervention study periods.

	Coef.	Std. Err.	p	95% CI
BASELINE PERIOD				
Constant	-3,349	0,220	0,000	[-3,779; -2,918]
Intervention	-0,101	0,105	0,333	[-0,307; 0,104]
Gender	-0,559	0,112	0,000	[-0,779; -0,340]
Involuntary	1,051	0,105	0,000	[0,844; 1,257]
Age	-0,013	0,004	0,000	[-0,020; -0,006]
Comorbid F1	-0,250	0,137	0,068	[-0,519; 0,018]
Dg F0	1,050	0,184	0,000	[0,689; 1,412]
Dg F1	0,742	0,168	0,000	[0,412;1,072]
Dg F2	-0,160	0,144	0,265	[-0,442; 0,122]
Dg F6	0,515	0,305	0,091	[-0,082; 1,113]
Comorbid F6	0,198	0,165	0,231	[-0,125; 0,520]

INTERVENTION PERIOD				
Constant	-3,441	0,251	0,000	[-3,932; -2,950]
Intervention	-0,648	0,124	0,000	[-0,890; -0,406]
Gender	-0,183	0,119	0,125	[-0,417; 0,051]
Involuntary	1,335	0,120	0,000	[1,100; 1,569]
Age	-0,015	0,004	0,000	[-0,023; -0,007]
Comorbid F1	0,028	0,143	0,842	[-0,251; 0,308]
Dg F0	0,679	0,203	0,001	[0,282; 1,077]
Dg F1	0,386	0,184	0,036	[0,025; 0,748]
Dg F2	-0,522	0,152	0,001	[-0,819; -0,224]
Dg F6	1,479	0,282	0,000	[0,926; 2,031]
Comorbid F6	0,165	0,165	0,316	[-0,157; 0,487]

Supplement Table 5. The results of the regression analysis on the number of physical restraints due to aggression during baseline and intervention study periods.

	Coef.	Std. Err.	p	95% CI
BASELINE PERIOD				
Constant	-3,727	0,255	0,000	[-4,227; -3,227]
Intervention	-0,028	0,120	0,817	[-0,264; 0,208]
Gender	-0,387	0,127	0,002	[-0,636; -0,138]
Involuntary	1,064	0,122	0,000	[0,825; 1,304]
Age	-0,014	0,004	0,000	[-0,022; -0,006]
Comorbid F1	-0,056	0,153	0,716	[-0,355; 0,244]
Dg F0	0,905	0,219	0,000	[0,475; 1,335]
Dg F1	0,710	0,195	0,000	[0,328; 1,091]
Dg F2	-0,176	0,163	0,278	[-0,495; 0,142]
Dg F6	0,461	0,349	0,186	[-0,222; 1,144]
Comorbid F6	0,323	0,184	0,079	[-0,038; 0,683]
INTERVENTION PERIOD				
Constant	-3,991	0,289	0,000	[-4,557; -3,425]
Intervention	-0,936	0,145	0,000	[-1,221; -0,651]
Gender	-0,248	0,136	0,069	[-0,515; 0,019]
Involuntary	1,235	0,135	0,000	[0,971; 1,499]
Age	-0,012	0,005	0,011	[-0,021; -0,003]
Comorbid F1	0,054	0,160	0,736	[-0,260; 0,368]
Dg F0	0,723	0,240	0,003	[0,252; 1,193]
Dg F1	0,539	0,215	0,012	[0,117; 0,961]
Dg F2	-0,211	0,176	0,233	[-0,556; 0,135]
Dg F6	1,659	0,324	0,000	[1,024; 2,294]
Comorbid F6	0,124	0,189	0,513	[-0,247; 0,494]

Supplement Table 6. The results of the regression analysis on the number of physical restraints in the experimental and control groups.

	Coef.	Std. Err.	p	95% CI
EXPERIMENTAL GROUP				
Constant	-3,842	0,288	0,000	[-4,407; -3,276]
Study period	-0,559	0,127	0,000	[-0,808; -0,309]
Gender	-0,455	0,134	0,001	[-0,718; -0,193]
Involuntary	1,158	0,139	0,000	[0,884; 1,431]]
Age	-0,005	0,005	0,265	[-0,014; 0,004]
Comorbid F1	0,273	0,155	0,078	[-0,030; 0,576]
Dg F0	0,433	0,240	0,071	[-0,038; 0,903]
Dg F1	0,690	0,216	0,001	[0,267; 1,113]
Dg F2	-0,106	0,178	0,550	[-0,455; 0,243]
Dg F6	1,610	0,331	0,000	[0,962; 2,259]
Comorbid F6	0,363	0,180	0,044	[0,010; 0,716]
CONTROL GROUP				
Constant	-3,305	0,200	0,000	[-3,696; -2,913]
Study period	0,020	0,093	0,834	[-0,163; 0,202]
Gender	-0,304	0,103	0,003	[-0,506; -0,101]
Involuntary	1,108	0,095	0,000	[0,922; 1,295]
Age	-0,019	0,003	0,000	[-0,026; -0,013]
Comorbid F1	-0,321	0,128	0,012	[-0,572; -0,070]
Dg F0	1,264	0,167	0,000	[0,937; 1,590]
Dg F1	0,664	0,155	0,000	[0,361; 0,968]
Dg F2	-0,271	0,132	0,040	[-0,530; -0,012]
Dg F6	0,496	0,286	0,082	[-0,064; 1,056]
Comorbid F6	0,074	0,155	0,631	[-0,229; 0,377]

Supplement Table 7. The results of the regression analysis on the number of physical restraints due to aggression in the experimental and control groups.

	Coef.	Std. Err.	p	95% CI
EXPERIMENTAL GROUP				
Constant	-4,010	0,331	0,000	[-4,659; -3,361]
Study period	-0,767	0,152	0,000	[-1,065; -0,469]
Gender	-0,445	0,155	0,004	[-0,750; -0,141]
Involuntary	1,272	0,159	0,000	[0,961; 1,584]
Age	-0,009	0,005	0,082	[-0,020; 0,001]
Comorbid F1	0,278	0,178	0,118	[-0,070; 0,627]
Dg F0	0,033	0,309	0,914	[-0,572; 0,639]
Dg F1	0,634	0,248	0,011	[0,147; 1,121]
Dg F2	-0,146	0,202	0,468	[-0,542; 0,249]
Dg F6	1,678	0,366	0,000	[0,960; 2,395]
Comorbid F6	0,284	0,213	0,181	[-0,132; 0,701]
CONTROL GROUP				

Constant	-3,863	0,231	0,000	[-4,315; -3,411]
Study period	0,145	0,106	0,170	[-0,062; 0,352]
Gender	-0,238	0,116	0,041	[-0,465; -0,010]
Involuntary	1,038	0,108	0,000	[0,828; 1,249]
Age	-0,016	0,004	0,000	[-0,023; -0,008]
Comorbid F1	-0,111	0,141	0,429	[-0,387; 0,164]
Dg F0	1,302	0,188	0,000	[0,933; 1,672]
Dg F1	0,657	0,178	0,000	[0,308; 1,006]
Dg F2	-0,229	0,149	0,124	[-0,521; 0,063]
Dg F6	0,553	0,324	0,088	[-0,083; 1,189]
Comorbid F6	0,219	0,168	0,193	[-0,111; 0,549]

Supplement Table 8. The results of the regression analysis on the number of incidents (all groups and study periods).

	Coef.	Std. Err.	p	95% CI
AGGRESSIVE INCIDENTS				
Constant	-3,260	0,158	0,000	[-3,569; -2,950]
Study period	-0,169	0,071	0,017	[-0,308; -0,031]
Intervention	-0,517	0,076	0,000	[-0,666; -0.368]
Gender	-0,004	0,075	0,960	[-0,151; 0,143]
Involuntary	0,928	0,074	0,000	[0,783; 1,073]
Age	-0,012	0,003	0,000	[-0,017; -0,007]
Comorbid F1	0,022	0,090	0,811	[-0,156; 0,199]
Dg F0	0,580	0,138	0,000	[0,310; 0,850]
Dg F1	0,519	0,119	0,000	[0,285; 0,752]
Dg F2	-0,159	0,095	0,094	[-0,345; 0.027]
Dg F6	0,790	0,194	0,000	[0,409; 1.170]
Comorbid F6	0,407	0,104	0,000	[0,202; 0.612]
PHYSICAL RESTRAINT				
Constant	-3,362	0,169	0,000	[-3,694; -3,031]
Study period	-0,175	0,075	0,020	[-0,323; -0,027]
Intervention	-0,316	0,080	0,000	[-0,472; -0,159]
Gender	-0,377	0,082	0,000	[-0,538; -0,216]
Involuntary	1,136	0,079	0,000	[0,982; 1,291]
Age	-0,014	0,003	0,000	[-0,019; -0,009]
Comorbid F1	-0,124	0,099	0,210	[-0,319; 0,070]
Dg F0	0,948	0,139	0,000	[0,676; 1,221]
Dg F1	0,666	0,126	0,000	[0,419; 0,913]
Dg F2	-0,202	0,106	0,057	[-0,411; 0,006]
Dg F6	0,774	0,218	0,000	[0,347; 1,200]
Comorbid F6	0,202	0,117	0,083	[-0,026; 0,431]
